# Dynamic UAV Deployment Scheme Based on Edge Computing for Forest Fire Scenarios

**DOI:** 10.3390/s24134337

**Published:** 2024-07-04

**Authors:** Weihao Zuo, Yongju Xian

**Affiliations:** School of Communications and Information Engineering, Chongqing University of Posts and Telecommunications, Chongqing 400065, China

**Keywords:** forest fire, UAV, edge computing, deep reinforcement learning

## Abstract

This study investigates the dynamic deployment of unmanned aerial vehicles (UAVs) using edge computing in a forest fire scenario. We consider the dynamically changing characteristics of forest fires and the corresponding varying resource requirements. Based on this, this paper models a two-timescale UAV dynamic deployment scheme by considering the dynamic changes in the number and position of UAVs. In the slow timescale, we use a gate recurrent unit (GRU) to predict the number of future users and determine the number of UAVs based on the resource requirements. UAVs with low energy are replaced accordingly. In the fast timescale, a deep-reinforcement-learning-based UAV position deployment algorithm is designed to enable the low-latency processing of computational tasks by adjusting the UAV positions in real time to meet the ground devices’ computational demands. The simulation results demonstrate that the proposed scheme achieves better prediction accuracy. The number and position of UAVs can be adapted to resource demand changes and reduce task execution delays.

## 1. Introduction

Forests are crucial to the Earth’s ecosystems and human society, playing a vital role in maintaining ecological balance and offering essential ecological services. However, forest fires significantly impact human society and the economy. Thus, enhancing forest fire monitoring and emergency response speeds is crucial to reducing disaster losses and protecting the ecological environment. The use of unmanned aerial vehicles (UAVs) for forest fire emergency response is increasingly favored due to their high mobility, rapid deployment, and low cost [1].

UAVs offer rapid, high-resolution, wide-area mapping [2,3] and can perform tasks such as data collection [4,5] and emergency communication [6]. After a natural disaster, large vehicle-mounted base stations often cannot be deployed to the disaster area immediately, preventing task offloading to the base station for processing [7]. UAVs are widely used in forestry for the accurate detection of resources [8], compensating for the shortcomings of traditional management methods.

Recently, with the rapid development of mobile edge computing (MEC) technology, the deployment of UAVs in forest fire scenarios has emerged as a research hotspot. Combining edge computing with UAV technology [9,10] enables efficient forest fire rescue and timely fire suppression measures, minimizing the fire spread and damage.

Currently, research on MEC server deployment has become a prominent task in the industry. Numerous scholars and engineers are dedicated to optimizing MEC server deployment to meet diverse application scenarios while ensuring service quality. Existing research on edge server deployment primarily falls into two categories: the operator’s perspective and the user’s perspective.

MEC server deployment is studied from the perspective of MEC operators. The author of [11] studied the MEC server deployment optimization problem from the MEC operator’s perspective and proposed a revenue maximization problem. A three-tier optimization algorithm was designed to maximize the total revenue of the MEC system. The author of [12] studied the problem of MEC server deployment from the perspective of a service provider. MEC server deployment and task scheduling were jointly optimized to maximize the service provider’s overall profit. The author of [13] studied the edge server deployment problem in 5G scenarios, formulated the problem as a profit optimization problem, and proposed an algorithm based on particle swarm optimization to solve the problem. Zhang et al. [14] formulated the edge server and service deployment problem by considering the number of MEC servers and its relationship with the base stations, storage capacity, and computational power, aiming to maximize the overall profit. They proposed a two-step approach, incorporating a clustering algorithm and nonlinear programming, to solve the problem.

The aim of the above research was to maximize the profit of the operator. However, from the user’s perspective, edge server deployment must consider the user experience quality.

Thus, MEC server deployment has also been studied from the user’s perspective. Ling et al. [15] initially utilized a network traffic prediction model based on graph convolutional networks to generate a network traffic distribution. They then formulated the edge server deployment problem as an optimization problem focused on delay and energy consumption, solving it with a particle swarm optimization algorithm. In [16], the author minimized the average service delay by jointly considering server placement, capacity allocation, and user offloading decisions. A genetic algorithm was designed to solve this combined optimization problem. The aforementioned research has investigated MEC server deployment from the user’s perspective and improved the quality of the user’s experience.

In UAV edge computing, UAVs provide edge computing services to users by deploying around them. Numerous studies have focused on UAV deployment.

Song et al. [17] formulated two stochastic games to decompose the minimization problem, aiming to reduce the system-wide computation costs for multi-user computation offloading and edge server deployment. Ning et al. [18] proposed a two-tier optimization algorithm to reduce the task processing delay. In the upper tier, a differential evolutionary algorithm addresses UAV deployment. In the lower tier, a distributed deep neural network generates offloading decisions. Bose et al. [19] utilized UAVs to provide computing services to edge devices. To improve the service quality for edge devices, the optimal UAV hovering height was calculated to achieve the maximum coverage with the minimal outage probability. In [20], the author studied UAV deployment to minimize the average processing delay, using a weighted K-means method. Deng et al. [21] proposed a four-stage alternating iteration algorithm to enhance the computational efficiency of the end system.

The aforementioned research employs deep reinforcement learning (DRL) algorithms to address UAV deployment issues. DRL allows the UAV edge computing system to learn by interacting with the environment and adjusting its strategy based on rewards, thereby enabling autonomous learning and improvement.

Guo et al. [22] integrated digital twins into UAV edge computing networks to explore intelligent UAV deployment and resource allocation. They designed a digital twin-assisted UAV deployment strategy and proposed a deep Q-network (DQN)-based task offloading solution. Wu et al. [23] proposed an autonomous deployment strategy utilizing deep reinforcement learning to determine the optimal hovering positions of UAVs in each mission area. Chen et al. [24] investigated the resource allocation problem under system delay constraints, optimized UAV motion and user association using DRL, and derived a closed-form solution for the user transmit power. Zhao et al. [25] jointly optimized the UAV’s position, computational task allocation, and the communication resources to minimize the sum of the execution latency and energy consumption. Additionally, their paper investigates a cooperative multi-agent deep reinforcement learning framework to address the aforementioned optimization problems. Yan et al. [26] jointly optimized the UAV’s position and task offloading strategy under the constraints of a limited network bandwidth and UAV power. Considering the dynamic variability of task arrivals, they utilized a deep deterministic policy gradient algorithm to solve the above problem. Zhao et al. [27] proposed joint optimization problems such as resolution adjustment, offloading decisions, computational capability allocation, communication resource orchestration, and multi-UAV position deployment under the constraints of offloading security, system delays, and user experience quality, using a DRL algorithm to solve these issues. Wang et al. [28] introduced an innovative multi-agent path planning framework leveraging deep reinforcement learning, designed to minimize the UAV’s total energy consumption while efficiently offloading user equipment tasks.

The severity of forest fires typically changes dynamically, leading to varying demands for UAV computing resources. In the early stages of a fire, only a few UAVs may be required for monitoring and early warning. As the fire expands and spreads, more UAVs may be needed for rescue operations. Therefore, the number of UAVs and the deployment strategies must be flexibly adjusted according to the fire’s development stage and scale, to meet its dynamically changing needs.

In summary, most existing research optimizes UAV deployment under the assumption of a fixed number of UAVs, neglecting the issue of dynamically changing resource demands. Consequently, when the resource demand increases, a small number of UAVs may be insufficient to handle a large number of tasks. To address these issues, this paper considers the joint optimization of both the number and position of UAVs. This paper investigates the dynamic deployment of UAVs in forest fire scenarios and utilizes UAVs for tasks in fire scenarios. The number of UAVs, the positions of the UAVs, and the task offloading decision are jointly optimized to minimize task processing delays. Given the constantly changing resource requirements in real scenarios, the proposed framework adjusts the number of UAVs to meet the resource demands in a slow timescale, while the UAV positions are adjusted in real-time to optimize task offloading in a fast timescale. The main contributions of this paper are as follows.

(1) We investigate the dynamic deployment problem of multiple UAVs in forest fire scenarios and establish a UAV-assisted mobile edge computing network for such environments. Considering the changing resource requirements in this scenario, a two-timescale UAV dynamic deployment algorithm is designed to jointly optimize the number of UAVs, their positions, and task offloading, thereby minimizing the task execution delay. The slow timescale adjusts the number of UAVs, while the fast timescale addresses UAV position deployment and task offloading decisions, considering UAV obstacle avoidance and energy consumption constraints.
(2) The slow timescale considers changing resource requirements in the fire scenario and designs a future-oriented quantity optimization mechanism. The GRU network is utilized to predict future resource changes, dynamically adjusting the number of UAVs based on these predictions.
(3) The fast timescale addresses the minimization of UAV processing task delays and designs a UAV location deployment algorithm based on the TD3 network for UAV position and task offloading decisions.
(4) The simulation results show that the scheme proposed effectively reduces the task execution delay and adapts to the dynamic changes of the forest environment.

## 2. System Model

**Figure 1:**
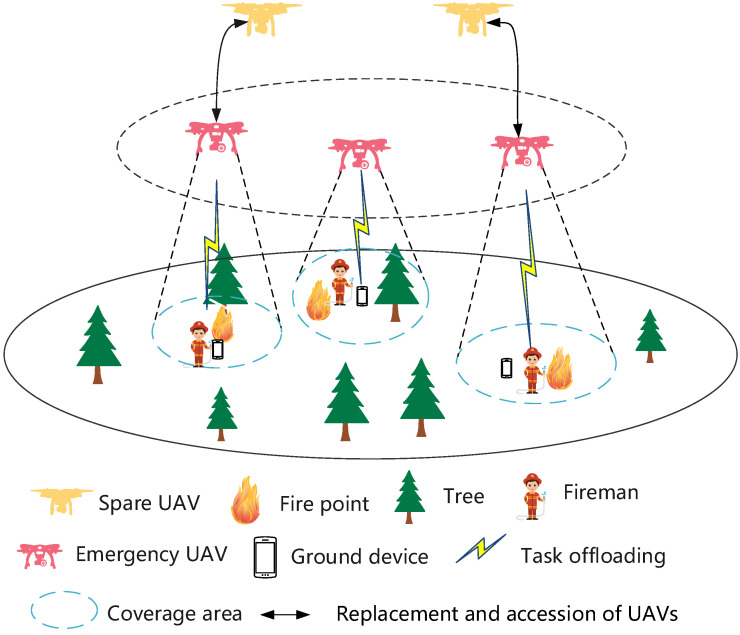
System model.

In this paper, we consider a UAV-assisted MEC network in a forest fire scenario. The system model diagram is shown in Figure 1, in which there are N UAVs deployed in the fire scenario and M ground devices. The UAVs are represented by the set N={1,2,…n,…N} and the ground devices by the set M={1,2,…,m,…M}. When the data collected by the ground devices require computation, the computational tasks can either be offloaded to the UAVs or performed locally. The service period of the UAV is discretely represented as T={1,2,…t,…T}, and the length of each time slot is denoted as δ.

The computational task generated by the ground device m in the time slot t is $I_{m}(t)=\left\{D_{m}(t),C_{m}(t),\tau_{m}(t)\right\}$, where $D_{m}(t)$ denotes the size of the task (bits), $C_{m}(t)$ denotes the computational resources required to compute each bit of the task(cycles/bit), and $\tau_{m}(t)$ denotes the delay tolerance threshold of the task. Define $\alpha_{m}(t)=\left\{\alpha_{m,1}(t),\alpha_{m,2}(t),\ldots ,\alpha_{m,n}(t),\ldots ,\alpha_{m,N}(t)\right\}$ as the task offloading vector of the ground device m, where $\alpha_{m,n}(t)\in \left\{0,1\right\}$ denotes the task offloading strategy of the ground device m in the time slot t. If $\alpha_{m,n}(t)=1$, the device m will offload the task to the UAV n for execution; if $\alpha_{m,n}(t)=0$, the device m will execute the task locally. Each ground device can offload the task to only one UAV per time slot.

### 2.1. Coordination and Communication Model

This paper utilizes line-of-sight transmission for the communication link between the ground devices and UAVs, with the UAV providing uniform bandwidth allocation to all devices. The position of the ground device m at time slot t is denoted as $P_{m}(t)=\left(x_{m}(t),y_{m}(t),0\right)$, and the position of the UAV n at time slot t is denoted as $O_{n}(t)=\left(x_{n}(t),y_{n}(t),h_{n}(t)\right)$. The distance between the UAV n and the ground device m is expressed in Equation (1):

(1) $$d_{m,n}(t)=\left\|P_{m}(t)-O_{n}(t)\right\|$$

Thus, the signal-noise ratio between the UAV n and the device m at time slot t is given by Equation (2):

(2) $${\mathrm{SINR}}_{m,n}(t)=\frac{p_{m}\beta_{0}}{{\left(d_{m,n}(t)\right)}^{2}\sigma^{2}}$$

where $\beta_{0}$ denotes the unit channel gain, $p_{m}$ denotes the transmit power of the ground device m, and $\sigma^{2}$ denotes the Gaussian white noise power of the wireless channel. According to Shannon’s formula, the transmission rate between the UAV n and the ground device m can be obtained, as shown in Equation (3):

(3) $$R_{m,n}(t)=B_{m}\log_{2}\left(1+{\mathrm{SINR}}_{m,n}(t)\right)$$

where $B_{m}$ denotes the channel bandwidth.

### 2.2. Task Offloading and Computation Model

When the task is executed on the local device, the local computation delay is as shown in Equation (4):

(4) $$t_{m}^{\mathrm{loc}}(t)=\frac{\left(1-\alpha_{m,n}(t))D_{m}(t)C_{m}(t\right)}{f_{m}}$$

where $f_{m}$ represents the computational capacity of the device m.

When the task is offloaded to a UAV with ample computing resources, the communication delay of the task m is as given by Equation (5):

(5) $$t_{m}^{\mathrm{trans}}(t)=\frac{\alpha_{m,n}(t)D_{m}(t)}{R_{m,n}(t)}$$

During the computation phase of the task, the computation delay of the UAV is as shown by Equation (6):

(6) $$t_{m}^{\mathrm{comp}}(t)=\frac{\alpha_{m,n}(t)D_{m}(t)C_{m}(t)}{f_{m,n}(t)}$$

where $f_{m,n}(t)$ denotes the computational resources allocated to the task m by the UAV n. Since the return result of the task is small, the return time of the result is ignored in this paper. Therefore, the delay of the task m is given by Equation (7):

(7) $$T_{m}(t)=t_{m}^{\mathrm{trans}}(t)+t_{m}^{\mathrm{comp}}(t)+t_{m}^{\mathrm{loc}}(t)$$

### 2.3. UAV Flight Model

In the UAV-assisted edge computing system, the UAV moves within the field. The position coordinate of the UAV n at time slot t is expressed as $O_{n}(t)=(x_{n}(t),y_{n}(t),h_{n}(t))$, and, at the next time slot t+1, it is expressed as $O_{n}(t+1)=(x_{n}(t+1),y_{n}(t+1),h_{n}(t+1))$. The movement model is shown in Equation (8):

(8) $$v_{n}(t)=\frac{\sqrt{{\left\|O_{n}(t+1)-O_{n}(t)\right\|}^{2}}}{\delta }$$

(9) $$v_{n}(t)\leq v_{\max}$$

The UAV moves within the target area, and its model is shown in Equations (10) and (11):

(10) $$0\leq y_{n}(t)\leq Y_{\max}$$

(11) $$0\leq x_{n}(t)\leq X_{\max}$$

where $X_{\max}$ and $Y_{\max}$ denote the range of the fire scenario, respectively.

To sustain the motion of the UAV, it consumes the corresponding propulsion energy, typically derived from its acceleration, deceleration, and steady flight phases. The UAV’s flight energy consumption impacts the edge computing system’s performance. According to the UAV propulsion model in [29], horizontal flight energy consumption is represented by Equation (12):

(12) $$E_{n}^{f}(t)=\delta (p_{0}(1+\frac{3{\left\|v_{n}(t)\right\|}^{2}}{v_{\mathrm{tip}}^{2}})+\frac{1}{2}d_{0}\rho_{0}sA{\left\|v_{n}(t)\right\|}^{3})+\delta (p_{i}{\left(\sqrt{1+\frac{{\left\|v_{n}(t)\right\|}^{4}}{4v_{0}^{4}}}-\frac{{\left\|v_{n}(t)\right\|}^{2}}{v_{0}^{4}}\right)}^{\frac{1}{2}})$$

When the UAV hovers, its flight speed is 0, i.e., $v_{n}(t)=0$, and the hovering energy consumption can be obtained by substituting $v_{n}(t)=0$ into Equation (12), as shown in Equation (13):

(13) $$E_{n}^{f}(t)=\delta \left(p_{i}+p_{0}\right)$$

To ensure the UAV’s safe return, the energy of the UAV is no less than zero when it returns to its point of departure, but, due to the different positions of the UAV when it returns, the energy of the UAV at this time is not the same. This paper models a UAV’s horizontal flight. For a more accurate analysis of the UAV return energy consumption, the vertical takeoff and landing energy consumption is examined next. This paper adopts the energy consumption model from [30]. The UAV’s vertical flight energy consumption is given by Equation (14):

(14) $$P_{\text{up-down}}(v_{n})=k_{1}mg\left(\frac{v_{n}}{2}+\sqrt{{\left(\frac{v_{n}}{2}\right)}^{2}+\frac{mg}{k_{2}^{2}}}\right)+c_{1}{\left(mg\right)}^{3/2}$$

where m denotes the mass of the UAV; g denotes the gravitational acceleration; $k_{1}$, $k_{2}$ and $c_{1}$ are the flight parameters of the UAV; and $k_{1}=0.8554$, $k_{2}=0.3051\sqrt{1/m}$ and $c_{1}=k_{1}/k_{2}$. $v_{n}$ is the takeoff and landing speed of the UAV, which is positive if the UAV takes off and negative if the UAV descends.

To minimize the UAV’s return trip energy consumption, this paper assumes that the UAV’s position during the return trip is $O_{n}^{end}=\left(x_{n}^{end},y_{n}^{end},h_{n}^{end}\right)$, with its starting point as $O_{\mathrm{start}}=\left(0,0,0\right)$. Assuming that the UAV returns at a fixed speed $v^{\mathrm{re}}$, the horizontal return time is obtained as in Equation (15):

(15) $$t_{n}^{\mathrm{ho}}=\frac{\sqrt{{\left(x_{n}^{end}\right)}^{2}+{\left(y_{n}^{end}\right)}^{2}}}{v^{\mathrm{re}}}$$

Thus, the horizontal return energy consumption of the UAV is given by Equation (16):

(16) $$E_{n}^{\mathrm{ho}}=t_{n}^{\mathrm{ho}}(p_{0}(1+\frac{3{\left\|v^{\mathrm{re}}\right\|}^{2}}{v_{\mathrm{tip}}^{2}})+\frac{1}{2}d_{0}\rho_{0}sA{\left\|v^{\mathrm{re}}\right\|}^{3})+t_{n}^{\mathrm{ho}}(p_{i}{\left(\sqrt{1+\frac{{\left\|v^{\mathrm{re}}\right\|}^{4}}{4v_{0}^{4}}}-\frac{{\left\|v^{\mathrm{re}}\right\|}^{2}}{v_{0}^{4}}\right)}^{\frac{1}{2}})$$

The descent energy consumption of the UAV’s return is shown in Equation (17):

(17) $$E_{n}^{\mathrm{ve}}=\frac{h_{n}^{end}}{v^{\mathrm{re}}}\left(k_{1}mg\left(\frac{v^{\mathrm{re}}}{2}+\sqrt{{\left(\frac{v^{\mathrm{re}}}{2}\right)}^{2}+\frac{mg}{k_{2}^{2}}}\right)\right)+\frac{h_{n}^{end}}{v^{\mathrm{re}}}\left(c_{1}{\left(mg\right)}^{3/2}\right)$$

In summary, the minimum energy consumption for the UAV’s return trip is as shown in Equation (18):

(18) $$E_{n}^{\mathrm{alert}}=E_{n}^{\mathrm{ho}}+E_{n}^{\mathrm{ve}}$$

The operational energy consumption of the UAV is shown in Equation (19):

(19) $$E_{n}(t)=E_{n}^{f}(t)+\sum_{m=1}^{M}t_{m}^{\mathrm{comp}}(t)\varepsilon_{n}{\left(f_{n,m}\left(t\right)\right)}^{3}$$

where $\varepsilon_{n}$ is the capacitance coefficient of the UAV and its value is $\varepsilon_{n}=10^{-28}$.

### 2.4. Obstacle Avoidance Model

In complex forest fire scenarios, the forest area is often in dangerous terrain with obstacles such as dangerous peaks and boulders, and dynamic obstacles such as the temperature and smoke at the fire site can also pose a threat to the flight of the UAV. Therefore, when modeling UAV flight in complex forest environments, it is crucial to account for obstacles to ensure efficient UAV operation. To prevent collisions between UAVs and dynamic obstacles like the temperature and smoke, the velocity of these obstacles is defined as $v^{\mathrm{obs}}(t)$, so the relative speed of the UAV and obstacles can be obtained as shown in Equation (20):

(20) $$v_{n}^{\mathrm{obs}}(t)=v_{n}(t)-v^{\mathrm{obs}}(t)$$

At this relative velocity, the obstacles remain stationary relative to the UAV. Thus, if the 2D position coordinates of the UAV n at time slot t are denoted as $O_{n}(t)=\left(x_{n}(t),y_{n}(t)\right)$, then, at the relative velocity, the coordinates at time slot t+1 can be expressed as $O_{n}(t+1)=\left(x_{n}(t)+v_{n}^{x}(t),y_{n}(t)+v_{n}^{y}(t)\right)$. The obstacle between the UAV n at time slot t and time slot t+1 is modeled as a circle, the radius of the circle is $R_{k}$, and the relative coordinate of the center of the circle is $\left(x_{k},y_{k}\right)$. To prevent collisions during flight, the distance between the UAV and the circle’s center must exceed the circle’s radius. The UAV’s flight trajectory can be derived using a linear equation, as shown in Equation (21):

(21) $$v_{n}^{y}(t)x+v_{n}^{x}(t)y+x_{n}(t)v_{n}^{y}(t)-y_{n}(t)v_{n}^{x}(t)=0$$

Thus, the distance from the circle’s center to the UAV’s flight path can be obtained, as shown in Equation (22):

(22) $$d_{n,k}=\frac{\left|v_{n}^{y}(t)x_{k}+v_{n}^{x}(t)y_{k}+x_{n}(t)v_{n}^{y}(t)-y_{n}(t)v_{n}^{x}(t)\right|}{\sqrt{{\left(v_{n}^{y}(t)\right)}^{2}+{\left(v_{n}^{x}(t)\right)}^{2}}}$$

If the distance from the circle’s center to the UAV’s flight path is less than the circle’s radius, a collision between the UAV n and the obstacle k will occur. To prevent this, ensure that $d_{n,k}>R_{k}$.

### 2.5. Optimization Problem Description

Given the UAV’s limited resources, this paper aims to maximize their efficiency by minimizing the task execution delay. This is achieved through the joint optimization of UAV positioning and task offloading decisions, as formulated in Equation (23):

(23) $$\begin{array}{l} P:\underset{O,\alpha }{\min}\frac{1}{TM}\sum_{t\in T}\sum_{m\in M}T_{m}(t)\\ s.t.\;C1:T_{m}(t)\leq \tau_{m}(t),\;\forall t\in T,\;\forall m\in M\\ \;C2:\alpha_{n,m}(t)\in \left\{0,1\right\},\;\forall n\in N,\;\forall m\in M\\ \;C3:\sum_{n=1}^{N}\alpha_{n,m}(t)\leq 1,\;\forall n\in N,\;\forall m\in M\\ \;C4:\sum_{m=1}^{M}f_{n,m}\leq f_{n}^{\max},\;\forall n\in N,\;\forall m\in M\\ \;C5:0\leq x_{n}(t)\leq X_{\max},\;0\leq y_{n}(t)\leq Y_{\max},\\ \;\;\forall n\in N,\;\forall t\in T\\ \;C6:d_{n,k}>R_{k},\;\forall n\in N,\;\forall k\in K \end{array}$$

where C1 denotes the computational delay constraint of the task, C2 signifies the offloading decision constraint for the ground devices, C3 indicates that the task of one device is offloaded to one UAV at most, C4 represents the computational resource constraint of the UAV $f_{n}^{\max}$, C5 denotes the activity range of the UAV, and C6 denotes the obstacle avoidance constraint of the UAV.

## 3. Two-Timescale UAV Dynamic Deployment Algorithm

**Figure 2:**
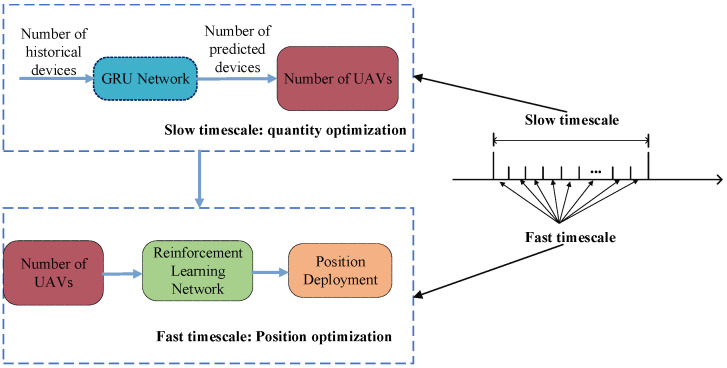
Two-timescale deployment algorithm structure.

In dynamically changing forest fire scenarios, UAVs must adapt accordingly. For instance, when the fire in an area is increasing or decreasing, the number of UAVs should also increase or decrease to adapt to the increase or decrease in the number of tasks in the fire scenario. Thus, both the quantity and positioning of the UAVs need to be continuously adjusted. Since the ground devices in forest fire scenarios continuously generate computational tasks, and the UAVs need to process these tasks and move in real time, the fast-timescale approach is adopted. Given that UAVs are relatively slow from liftoff to arrival at the target area, often taking minutes, changing the number of UAVs is considered a slow-timescale decision. Therefore, this paper designs a two-timescale UAV deployment framework, shown in Figure 2, which defines consecutive time slots T as a timeframe and denotes the timeframe k of the first timeframe as $T_{k}=\left\{kT,kT+1,\ldots ,(k+1)T-1\right\}$.

(1) Slow timescale: At the beginning of each timeframe, the number of UAVs is re-determined based on the predicted task requirements and the UAV’s energy.
(2) Fast timescale: In each time slot, the TD3 algorithm is utilized to make the offloading decision between the UAV’s movement and the ground devices based on the ground device’s task information and position and the UAV’s position information.

Accordingly, the two-timescale deployment algorithm makes decisions at different timescales, as illustrated in Algorithm 1. And, Algorithm 2 and Algorithm 3 are in Algorithm 1.
**Algorithm 1** Two-Timescale Deployment AlgorithmInput: Number of historical ground devices, UAV informationOutput: Number of UAVs deployed, UAVs’ positions, offloading decisions1. **for** t=1 to T **do**2.    **for** t=1, 1+ΔT,…, **do**3.    Execute the prediction algorithm to determine the task requirements at the fire scenario.4.    Get the number of UAVs using Algorithm 25.    **end for**6.    **for** UAV n∈N **do**7.       Execute Algorithm 3 to determine the positions of the UAVs and the offloading decisions.8.    **end for**9. **end for**

**Algorithm 2** Optimization algorithm for number of UAVs**Inputs:** Number of predicted ground devices, Initial UAV’s energy $E_{\mathrm{uav}}$, Minimum UAV energy requirement $E_{n}^{\mathrm{alert}}$
**Output:** Number of UAVs deployed1. **for** $T_{0}$, $T_{k}$,…, $T_{K-1}$ **do**2.    **while** predicted demand calculation > actual number of tasks provided **do**3.               $N_{T_{k}}+1$
4.    **end while**5.    **while** predicted demand calculation*1.2 < actual number of tasks provided **do**6.             
$N_{T_{k}}-1$
6.    Select the UAV with the least energy to execute $N_{T_{k}}-1$
7.    **end while**8.    **for** $n=1,2,\ldots ,N_{T_{k}}$ **do:**9.        **if** $E_{n}-\sum_{t=1}^{T}E_{n}(t)\leq E_{n}^{\mathrm{alert}}$ **do:**
10.            Lift a UAV to replace the UAV n.11.       **end if**12.    **end for**13. **end for**

**Algorithm 3** Reinforcement-learning-based UAV position deployment algorithm**Input:** Device position information P(t), UAV’s energy E(t), Task characteristics I(t)
**Output:** UAV’s position, Offloading decision **Initialization:** Initialize network parameters1. **for** t=1,2,3,…,T **do**2.       UAV performs an action based on the current state s(t), receives a reward r(t) and the next state s(t+1).3.       Store the tuple $\left(s(t),a(t),r(t),r(t+1)\right)$ in the experience pool.4.       Randomly sample a batch from the experience pool.5.       Calculate the loss function according to Equation (30) and update the parameters of the critic network with $\theta_{1}^{Q}$ and $\theta_{2}^{Q}$.6.       **if** t mod δ **do**7.             Update the $\theta^{\mu }$ parameters of the actor network according to Equation (31).8.             Update the parameters of the target network $\theta_{1}^{Q^{\prime }}$, $\theta_{2}^{Q^{\prime }}$ and $\theta^{\mu^{\prime }}$ according to Equations (32) and (33).9.       **end if**10. **end for**

### 3.1. Optimization Algorithm for the Number of UAVs

When UAVs are deployed, the number of UAVs can be determined based on the situation of the fire scenario and the energy of the UAVs to efficiently utilize the UAV resources. Deploying too many UAVs for small tasks at a fire scene can result in the waste of limited UAV resources. This paper does not predetermine the number of UAVs; instead, it determines the number to be dispatched based on the actual needs of the fire scenario.

**Figure 3:**
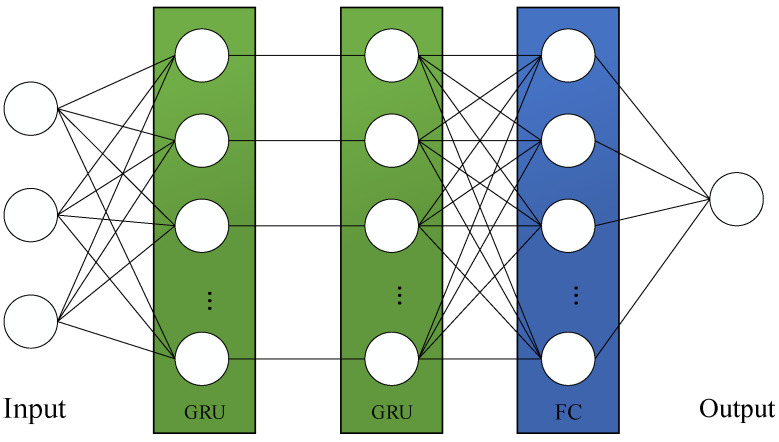
GRU forecasting model.

In this paper, the number of UAVs is determined by their computational power and the computational demand at the scenario. Firstly, this paper employs a GRU to learn the changes at the fire scene and predict the on-site resource demand. A GRU, a type of recurrent neural network, is more computationally efficient than the long short-term memory (LSTM) network and is therefore utilized in this study. The GRU network is used to predict the on-site resource demand, outputting predicted values based on previous changes in the fire scenario. The prediction model is illustrated in Figure 3.

In the actual deployment process, fixing the number of UAVs to a specific value may cause a “ping-pong effect,” leading to instability in the UAV numbers. Therefore, this paper permits a margin of error between the predicted and actual calculations, allowing the number of UAVs to remain stable within a certain range. In order to ensure system stability and accommodate fluctuations in the prediction errors and computational resource demands, we introduce the constant of 1.2 as a tolerance mechanism. This avoids frequent adjustments to the number of UAVs due to minor changes in demand, thereby enhancing the system’s reliability.

### 3.2. UAV Position Optimization Algorithm

The previous section addressed the optimization of the UAV numbers. This subsection focuses on solving the optimization problem to determine the UAV positions and task offloading decisions. Due to the time-varying nature of UAV positions, traditional algorithms struggle to meet the real-time requirements. Hence, we propose a TD3-based UAV position deployment algorithm, modeling the original optimization problem as a Markov decision process.

State space: The state space comprises information observed by the agent about the UAV and ground device, including the ground device’s position, the UAV’s energy, and the task characteristics, as shown in Equation (24):

(24) $$S(t)=\left\{P(t),E(t),I(t)\right\}$$

where $P(t)=\left\{P_{1}(t),P_{2}(t),\ldots ,P_{M}(t)\right\}$ denotes the ground device position set; $E(t)=\left\{E_{1}(t),E_{2}(t),\ldots ,E_{N}(t)\right\}$ denotes the UAV energy set; $I(t)=\left\{I_{1}(t),I_{2}(t),\ldots ,I_{M}(t)\right\}$ denotes the task characteristic set.

Action space: The action space comprises the UAV position and the task offloading decision, as shown in Equation (25):

(25) $$A(t)=\left\{O(t),\alpha_{m}(t)\right\}$$

where $O(t)=\left\{O_{1}(t),O_{2}(t),\ldots ,O_{N}(t)\right\}$ denotes the UAV position set.

Reward function: In DRL, the reward is the feedback from the environment to the agent for performing the action. The optimization objective function is thus transformed into the maximization of the cumulative reward through the design of the reward function. Based on the optimization problem presented in this paper, at each time slot, the agent executes an action based on the observed state, receives the corresponding reward, and generates the optimal decision by maximizing the reward value. To minimize the task execution delay, this paper sets the inverse of the objective function as the reward value, as shown in Equation (26):

(26) $$r(t)=\frac{1}{\sum_{m\in M}T_{m}(t)}$$

The TD3 algorithm is a DRL algorithm in the actor–critic framework. Firstly, to address the overestimation issue inherent in the deep deterministic policy gradient (DDPG), the TD3 algorithm integrates the concept of a double-deep Q-network. In TD3, two critic networks are employed, and the smaller value is used when calculating the target to prevent overestimation. Secondly, TD3 introduces a target policy smoothing technique to enhance the accuracy of the target value estimation, thereby ensuring training stability. Finally, the actor network in TD3 employs a delayed update technique, meaning that the critic network is updated multiple times before the actor network, thus reducing the cumulative error.

The TD3 algorithm will make decisions regarding UAV movement and offloading based on the device position information, the UAV’s computational capabilities, and the task characteristics. The actor network outputs and executes actions A(t) based on the environment state S(t). The critic network outputs the estimated reward value r(t) based on the environment state S(t) and the actions A(t). Finally, the environment state transitions to a new state S(t+1).

The target Q value in the TD3 algorithm is derived from the minimum of the two critic network outputs. The formula for the computation of this target value is given in Equation (27):

(27) $$y(t)=r(t)+\underset{i=1,2}{\min}Q(S(t+1),\bar{A}(t)\left|\theta_{i}^{Q}\right.)$$

As elaborated above, the TD3 algorithm incorporates target policy smoothing regularization. Target strategy smoothing regularization is mainly used to add noise to the actor network’s output, and the noise can play the role of regularization, which renders the update of the value function smoother and improves the accuracy of the estimated value. Consequently, the action above is as shown in Equation (28):

(28) $$\bar{A}(t)=\pi (S(t)|\theta^{\mu })+\bar{\varepsilon }$$

(29) $$\bar{\varepsilon }\sim clip(N(0,\sigma^{2}),-c,c)$$

where $\sigma^{2}$ denotes the noise strategy and c represents the noise threshold.

The critic network is trained by minimizing a loss function, defined as the mean square error (MSE) between the critic network’s estimate and the target value, as illustrated in Equation (30):

(30) $$L(\theta^{Q})=E\left[{\left(y(t)-Q(S(t),A(t)\left|\theta^{Q}\right.)\right)}^{2}\right]$$

The objective of updating the actor network is to maximize the expected return. Consequently, the actor network’s parameters are updated using the gradient shown in Equation (31):

(31) $$\nabla_{\theta^{\mu }}J=E\left[\nabla_{a}Q(S,A|\theta^{Q}){|}_{S=S(t),A=\mu (S(t)}\nabla_{\theta^{\mu }}\mu (S|\theta^{\mu }){|}_{S=S(t)}\right]$$

The target network is updated using a soft update mechanism, as shown in Equations (32) and (33):

(32) $$\theta_{i=1,2}^{Q^{\prime }}=\tau \theta_{i}^{Q}+(1-\tau )\theta_{i}^{Q^{\prime }}$$

(33) $$\theta^{\pi^{\prime }}=\tau \theta^{\pi }+(1-\tau )\theta^{\pi^{\prime }}$$

where τ denotes the learning rate, $\tau \in \left[0,1\right]$.

### 3.3. Algorithm Time Complexity Analysis

The two-timescale deployment algorithms proposed in this paper comprise the UAV number optimization algorithm on a slow timescale and the UAV position optimization algorithm based on reinforcement learning on a fast timescale. In the slow timescale, assuming that there are K timeframes, it is known from [31] that the parameters in the GRU can be reduced to two matrices, U and V, which can be mapped to the inputs and outputs, respectively. $F_{0}$ represents the dimension of the input layer. Let H denote the number of neurons in the GRU layer; the dimension of U is $HF_{0}$, and the dimension of V is $H^{2}$. Additionally, these two matrices U and V are required by the GRU network to learn; the total dimension of the GRU is $3(HF_{0}+H^{2}+H)$ and the time complexity of the algorithm can be expressed as $O\left(3K(HF_{0}+H^{2}+H)\right)$.

In the fast timescale, assuming that the actor network and the critic network have $Z_{a}$ and $Z_{c}$ fully connected layers, respectively, the number of i neurons in the first layer is $F_{a,z}$ and $F_{c,z}$. Given that the TD3 algorithm employs two critic networks, the complexity of the actor network is $\sum_{z=0}^{Z_{a}}F_{a,z}F_{a,z+1}$, and the complexity of the critic network is $2\sum_{z=0}^{Z_{c}}F_{c,z}F_{c,z+1}$. Therefore, the time complexity of the UAV position deployment algorithm is $O\left(\sum_{z=0}^{Z_{a}}F_{a,z}F_{a,z+1}+2\sum_{z=0}^{Z_{c}}F_{c,z}F_{c,z+1}\right)$. The overall time complexity of the algorithm is shown in Equation (34):

(34) $$\begin{array}{l} O\left(\sum_{z=0}^{Z_{a}}F_{a,z}F_{a,z+1}+2\sum_{z=0}^{Z_{c}}F_{c,z}F_{c,z+1}\right)+O\left(3K(HF_{0}+H^{2}+H)\right)\\ =O\left(\sum_{z=0}^{Z_{a}}F_{a,z}F_{a,z+1}+\sum_{z=0}^{Z_{c}}F_{c,z}F_{c,z+1}+H^{2}\right) \end{array}$$

## 4. Simulation and Analysis

In this section, the proposed scheme is simulated and its performance is analyzed.

### 4.1. Simulation Parameter Setting

To verify the performance of the proposed scheme, we utilize Python 3.8.0 and TensorFlow2.7.0 for simulation on the Windows platform. We set the target area to a size of 1000×1000 m^2^, containing multiple UAVs, several ground devices, and seven fire points. The initial positions of the UAVs are $\left(0,0,0\right)$, while the ground devices and fire points are randomly distributed within the target area. The UAVs are airborne, and the computational tasks generated by the ground devices can either be processed locally or offloaded to the UAVs for computation. The sizes of input data $D_{n}(t)$ are randomly generated within [2, 4] Mbits, and the number of CPU cycles $C_{n}(t)$ is uniformly chosen from [100, 200]. To validate the effectiveness of the proposed scheme, it is compared with the DDPG, DQN, and Greedy algorithms. The specific parameter settings are detailed in Table 1 [25,28,30]. The proposed TD3 framework has two hidden-layer neural networks with 400 and 300 neurons. The learning rate and discount rate are set to 0.001 and 0.98, respectively. Moreover, the mini-batch size of the training samples is set to 64 and the optimizer is Adam. The GRU framework has two hidden-layer neural networks with 128 and 64 neurons. For the GRU framework, the learning rate is set at 0.01, and the Adam optimizer is employed.

### 4.2. Simulation Result Analysis

**Figure 4:**
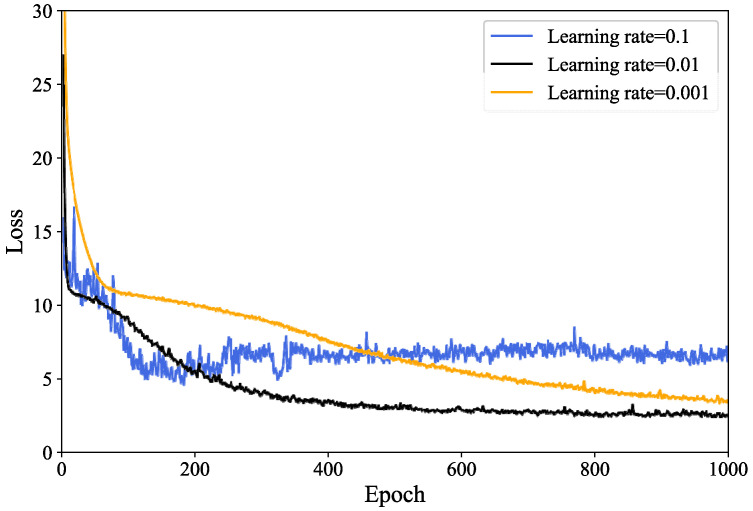
Loss value of the algorithm for different learning rates.

To verify the convergence of the algorithm, Figure 4 illustrates the variations in the loss function across different learning rates. As shown in Figure 4, although the value of the loss function varies with the different learning rates, they all eventually converge, demonstrating the algorithm’s feasibility. With an increase in the learning rate, the algorithm’s convergence accelerates and the parameter updates become more frequent, resulting in sharp fluctuations and potential divergence during training. This phenomenon occurs because a larger learning rate makes the algorithm more exploratory, whereas a smaller learning rate results in slower convergence and necessitates more iterations to achieve satisfactory results.

**Figure 5:**
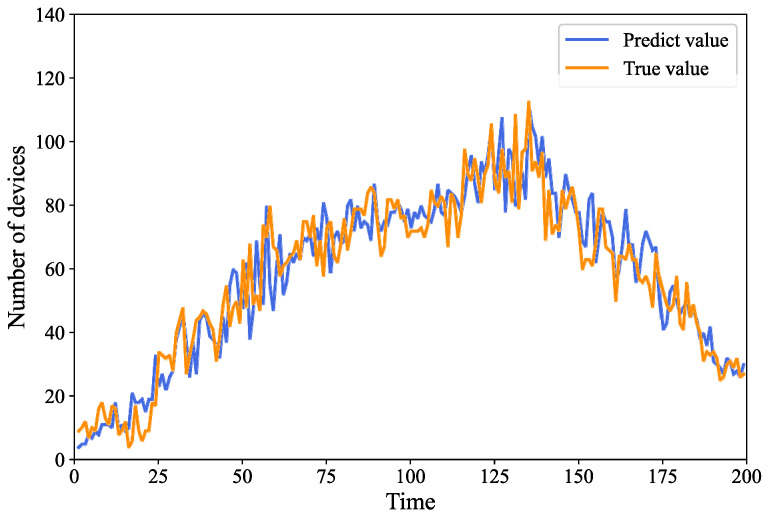
Equipment prediction results.

Figure 5 shows the prediction results of the devices. From the figure, it can be observed that the predicted values obtained using the GRU-based prediction algorithm proposed in this paper strongly overlap with the actual values, indicating a superior fitting effect. This further validates the effectiveness and accuracy of the GRU network for the device prediction task.

**Figure 6:**
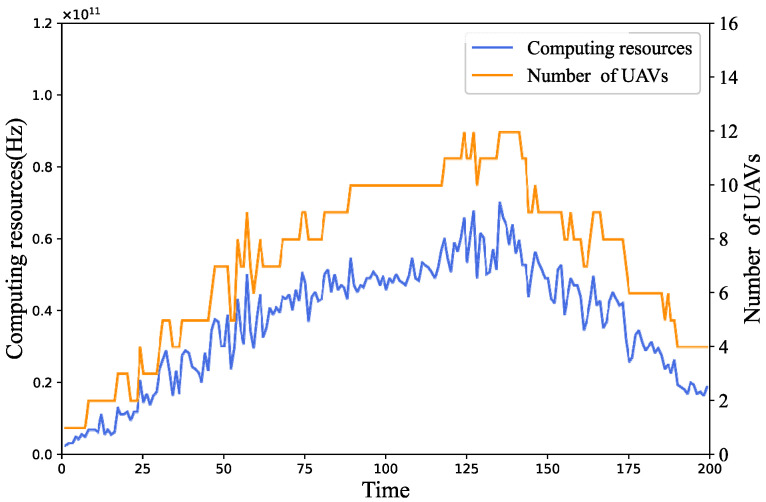
Changes in UAVs and computing resource requirements.

Figure 6 illustrates the relationship between the number of UAVs and the number of computational resources required by the ground devices. From the figure, it is apparent that as the computational resources required by the ground devices vary, the number of UAVs also exhibits a corresponding trend. This change reflects the close correlation between the demand for computing resources for the ground devices and the number of UAVs. Specifically, large fluctuations in the computing resources required by the ground devices lead to corresponding adjustments in the number of UAVs. When the fluctuations in the computing resources required by the ground devices are small, the number of UAVs remains unchanged, preventing constant adjustment.

**Figure 7:**
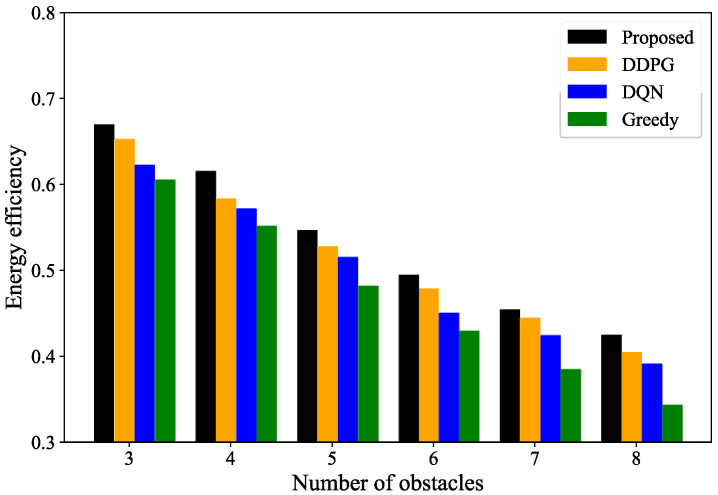
UAV energy efficiency for different numbers of obstacles.

Figure 7 shows the energy utilization of the UAVs with varying numbers of obstacles, assuming a fixed number of UAVs. As shown in the figure, the energy rate of the UAVs gradually decreases as the number of obstacles increases. This is because, with more obstacles, UAVs need to take longer paths to avoid them, thereby increasing their energy consumption. However, it can be seen from the figure that the UAV energy utilization for the scheme in this paper is consistently higher than that in the other schemes.

**Figure 8:**
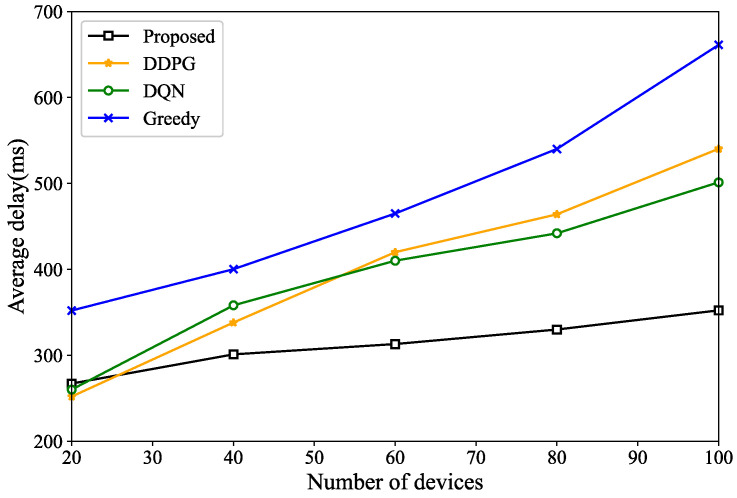
Average task execution latency for different numbers of devices.

Figure 8 shows the average delay variation of the tasks without considering the number of devices. From Figure 8, when the number of devices is small, the difference in the average execution delay of the tasks is minimal. As the number of devices increases, the average delay of the tasks in the proposed scheme remains relatively constant. This stability is due to the corresponding increase in the number of UAVs, ensuring that the shared computational resources remain sufficient. Compared to other schemes, the proposed scheme effectively adapts to the increase in the number of devices by considering the dynamic adjustment of the UAV numbers, whereas other schemes struggle to cope with such changes.

## 5. Conclusions

In forest fire scenarios, the UAVs’ positions and numbers are adjusted to accommodate the changing resource requirements in the field. Additionally, this study considers the change in energy during UAV service, enhancing the UAVs’ efficiency by replacing those with lower energy levels. Simultaneously, the obstacle problem during UAV flight is addressed by modeling obstacles and designing avoidance constraints. The task processing delay is minimized through the joint optimization of the UAV number, position, and offloading decisions. Finally, a two-timescale UAV dynamic deployment algorithm is proposed. In the slow timescale, the GRU network predicts future resource changes in the fire scenario, enabling the dynamic adjustment of the UAV numbers. In the fast timescale, a TD3 network-based algorithm is designed for UAV position deployment and task offloading decisions. The simulation results demonstrate that the proposed scheme effectively adapts to dynamic resource changes and significantly reduces the task processing delay.

Future research can further explore the co-optimization strategy of energy harvesting and UAV edge computing to achieve intelligent decision-making for optimal energy utilization and task scheduling, providing a more sustainable and efficient solution for forest fire rescue work.

## Figures and Tables

**Table 1:** Simulation parameter table.

Parameter	Value
UAV speed $v_{n}(t)$ (m/s)	0–30
UAV energy $E_{uav}$ (KJ)	120
Computing resources per UAV $f_{u}$ (GHz)	5
Gaussian noise power $\sigma^{2}$ (dBm)	−100
Channel gain $\beta_{0}$ (dB)	−50
Bandwidth B (MHz)	10
Size of task $D_{n}(t)$ (Mbits)	[2, 4]
Required CPU cycles per bit $C_{n}(t)$ (cycles/bit)	[100, 200]
Transmission power $p_{m}$ (W)	0.1
Weight per UAV m (Kg)	2
Blade profile power in hover $p_{0}$ (W)	79.86
Induced power in hover $p_{i}$ (W)	88.63
Tip speed of rotor blade $\nu_{\mathrm{tip}}$ (m/s)	120
Fuselage drag ratio $d_{0}$	0.6
Air density $\rho_{0}$ (kg/m^3^)	1.225
Rotor solidity S (m^3^)	0.05
Rotor disc area A (m^2^)	0.503
Mean rotor-induced velocity in hover $\nu_{0}$ (m/s)	4.03

## Data Availability

Data are contained within the article.
